# The Role of Indoleamine-2,3-Dioxygenase in Cancer Development, Diagnostics, and Therapy

**DOI:** 10.3389/fimmu.2018.00151

**Published:** 2018-01-31

**Authors:** Lilla Hornyák, Nikoletta Dobos, Gábor Koncz, Zsolt Karányi, Dénes Páll, Zoltán Szabó, Gábor Halmos, Lóránt Székvölgyi

**Affiliations:** ^1^MTA-DE Momentum Genome Architecture and Recombination Research Group, Department of Biochemistry and Molecular Biology, Faculty of Medicine, University of Debrecen, Debrecen, Hungary; ^2^Department of Biopharmacy, Faculty of Pharmacology, University of Debrecen, Debrecen, Hungary; ^3^Department of Immunology, Faculty of Medicine, University of Debrecen, Debrecen, Hungary; ^4^Department of Internal Medicine, Faculty of Medicine, University of Debrecen, Debrecen, Hungary; ^5^Department of Emergency Medicine, Faculty of Medicine, University of Debrecen, Debrecen, Hungary

**Keywords:** indoleamine-2,3-dioxygenase, gene expression, metabolism, immunotherapy, immune surveillance, cancer diagnostics, clinical trial

## Abstract

Tumors are composed of abnormally transformed cell types and tissues that differ from normal tissues in their genetic and epigenetic makeup, metabolism, and immunology. Molecular compounds that modulate the immune response against neoplasms offer promising new strategies to combat cancer. Inhibitors targeting the indoleamine-2,3-dioxygenase 1 enzyme (IDO1) represent one of the most potent therapeutic opportunities to inhibit tumor growth. Herein, we assess the biochemical role of IDO1 in tumor metabolism and immune surveillance, and review current diagnostic and therapeutic approaches that are intended to increase the effectiveness of immunotherapies against highly aggressive and difficult-to-treat IDO-expressing cancers.

## Introduction

### Biochemistry and Regulation of Indoleamine-2,3-Dioxygenase 1 (IDO1) Activity

**Figure 1:**
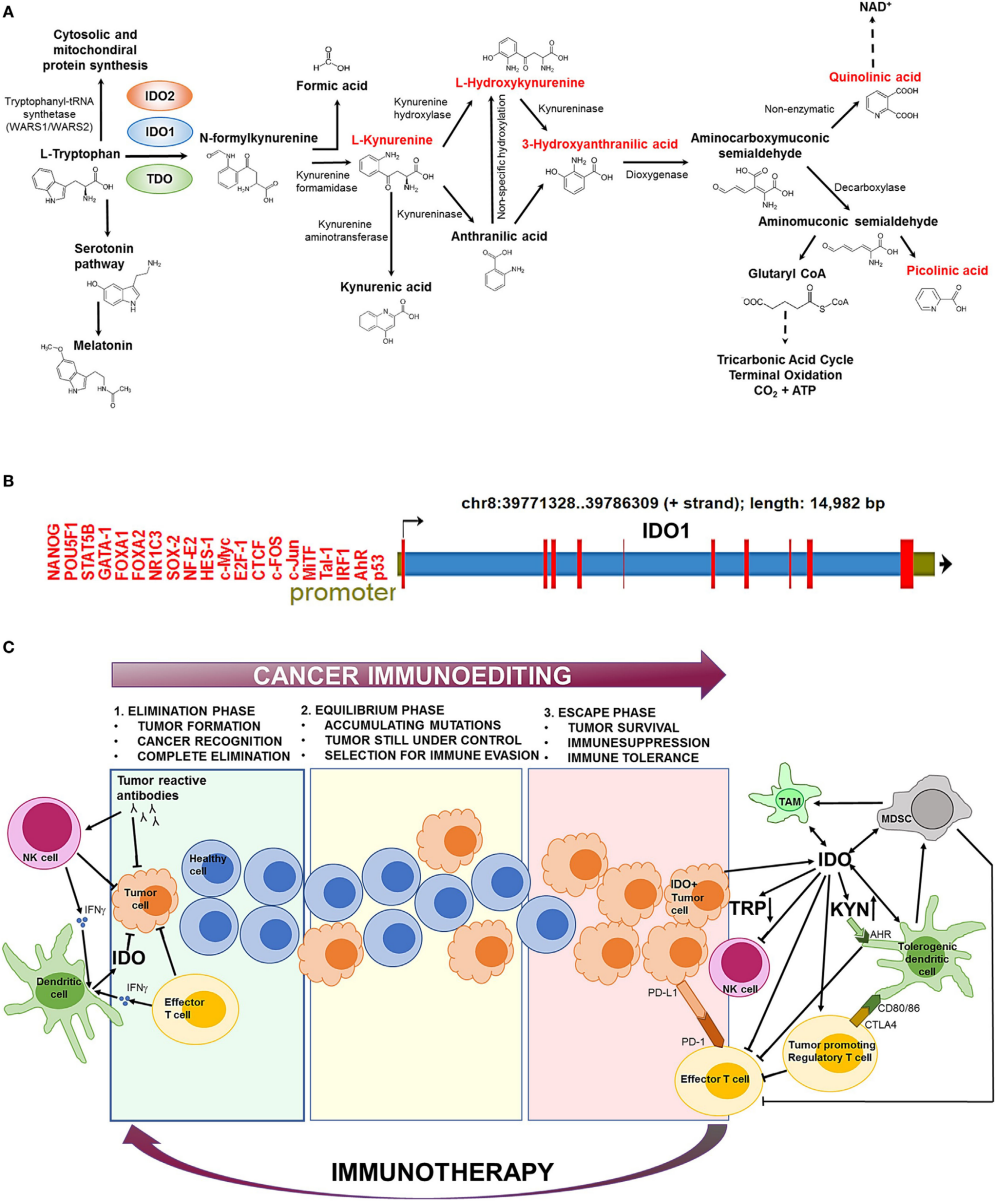
The biochemical function and regulation of indolamine-2,3-dioxygenase 1 (IDO1). **(A)** The kynurenine (Kyn) pathway of tryptophan (Trp) catabolism. l-Trp is metabolized in three separate biochemical pathways (indicated by arrows). In the Kyn pathway, IDO1/IDO2 and tryptophan-2,3-dioxygenase (TDO) catalyze the first and rate-limiting step of Trp degradation that gives rise to *N*-formylkynurenine. *N*-formylkynurenine is then transformed into l-Kyn and formic acid by kynurenine formamidase. l-Kyn is converted to anthranilic acid by kynureninase or l-hydroxykynurenine by kynurenine hydroxylase. Non-specific hydroxylation of anthranilic acid results in l-hydroxykynurenine. Kynureninase converts l-hydroxykynurenine to 3-hydroxyanthranilic acid that is further metabolized by hydroxyanthranilate dioxygenase to aminocarboxymuconic semialdehyde. The semialdehyde spontaneously forms quinolinic acid that is a precursor of NAD^+^ synthesis, or a decarboxylase enzyme converts it to aminomuconic semialdehyde. Aminomuconic semialdehyde is then converted to picolinic acid or glutaryl-CoA that is metabolized in the tricarbonic acid cycle and terminal oxidation. Metabolites that are highlighted in red have been directly implicated in immunosuppressive mechanisms and cancer development. **(B)** The structure of the IDO1 gene. IDO1 is located on chromosome 8 [39771328–39786309 forward (+) strand; 14,982 base pairs] comprising 10 exonic region (red bars). The promoter region (green section upstream the transcription start site) contains several transcription factor-binding sites that have been identified by ChIP sequencing. ChIP peaks were collected from the GTRD database of transcription binding sites (3). Only normal (non-transformed) cell types were considered. **(C)** The role of IDO1 in cancer immunoediting. In the first phase of immunoediting (“elimination”), sporadically arising transformed cells are destroyed by the innate and adaptive immune systems. Activated B cells produce tumor reactive antibodies to eradicate most transformed cells. Natural killer (NK) cells and effector T cells release inflammatory cytokines, such as IFN-γ, which activate dendritic cells (DCs) that secrete low levels of IDO1. IDO1 depletes the essential amino acid Trp from the tumor microenvironment that inhibits tumor growth. In the “equilibrium” phase, surviving tumor cells are still controlled by the immune system; however, they rapidly accumulate mutations. When the immune system can no longer block the abnormal and autonomous growth of “edited” cells, the tumor becomes clinically manifested (“escape”). The escape phase is associated with high IDO1 level that is primarily produced by tumor cells and tolerogenic immune cells [e.g., tolerogenic DCs, myeloid-derived suppressor cells (MDSCs), tumor-associated macrophages (TAMs)]. Trp depletion and Kyn accumulation lead to immunosuppression and tolerogenicity by inhibiting effector T cell and NK cell functions and stimulating regulatory T cells. IDO1 also promotes the expansion and activation of MDSCs and induces polarization of macrophages to a tolerogenic phenotype. Increased Kyn levels activate the aryl hydrocarbon receptor (AhR) that switch the activity of DCs from immunogenic to tolerogenic. Elevated CTLA4 expression of regulatory T cells results in further increase of IDO1 secretion by DCs. IDO1-induced expansion and activation of regulatory T cells, tolerogenic DCs, and MDSCs suppress the activity of antitumor effector T cells. Other immunosuppressive agents (e.g., PD-L1/PD-1, CTLA4) also inhibit effector T cell functions. Oncological immunotherapy aims to reverse immunoediting (backward arrow) by inhibiting and activating local immunosuppressive and tumor eradication mechanisms, respectively.

Indolamine-2,3-dioxygenase 1 is a cytosolic enzyme with a heme (Fe^2+^) prosthetic group that catalyzes the first and rate-limiting step of tryptophan (Trp) catabolism (Figure 1A). IDO1 converts the essential amino acid Trp to kynurenine (Kyn) by cleaving the 2,3-double bond of the indole ring while a molecular oxygen (O_2_) merges into the unsealed molecule. The product is *N*-formylkynurenine that becomes rapidly and spontaneously transformed into Kyn (1). In the next steps, Kyn is further converted to other active metabolites, such as hydroxykynurenine, anthranilic acid, kynurenic acid, 3-hydroxyanthranilic acid, quinolinic acid, and picolinic acid (Figure 1A). The two end-products of the pathway are NAD^+^ and ATP that both fuel cellular metabolism (2).

In humans, IDO1 has an evolutionary paralog (indolamine-2,3-dioxygenase 2; IDO2) and a functional ortholog (tryptophan-2,3-dioxygenase; TDO) that catalyze the same biochemical reaction; however, IDO2 and TDO show high tissue specificity and much lower expression level than IDO1 that significantly restrict their activity (2, 4). TDO is transcribed only in the liver^1^ [protein-level expression is not established (5)] and its major function is to control the free Trp concentration of the blood (4). IDO2 mRNA is expressed at low levels in the placenta and liver (protein expression is not known^2^), while IDO1 shows a high protein expression in the peripheral lymph organs (lymph nodes, spleen and tonsils^3^).

The activity of IDO1 is mainly regulated at the transcriptional level (Figure 1B). *Bona fide* transcription factor binding sites have been detected by ChIP-seq for a large catalog of human transcription factors (3). The identified gene regulatory proteins involve (i) NF- κB, which allows IDO1 mRNA expression regulation through the interferon pathway (6), (ii) the aryl hydrocarbon receptor (AhR) that binds to putative dendritic-cell responsive elements and promotes the l-Kyn-dependent induction of IDO1 (7, 8), and (iii) CTCF that mediates IDO1 expression *via* long-range chromatin looping interactions between the promoter and distal enhancer regions (9).

Superimposed on the transcriptional control of IDO1, specific posttranslational mechanisms also operate that affect the activity and half-life of the enzyme. For instance, the diffusible messenger nitrogen monoxide (NO) reacts with the heme cofactor of IDO1 generating ferric (Fe^3+^) heme and nitrate (NO_3_), which leads to the dose-dependent and reversible inhibition of enzymatic activity (10, 11). It has been also shown that endogenous NO production accelerates the proteasomal degradation of IDO1 (12). Other antioxidants like the anti-inflammatory agent pyrrolidine dithiocarbamate (13) restrict the availability of heme and thereby block holoenzyme assembly (14). In inflammatory conditions, NO and superoxide anions $\left({\text{O}}_{2}^{-}\right)$ are simultaneously produced in large amounts, which rapidly generate the highly reactive peroxynitrite anion. Peroxynitrite inhibits IDO1 *via* the nitration of critical tyrosine residues (Tyr15, Tyr345, and Tyr353), without affecting IDO1 protein level (15, 16).

Hypoxia also leads to reduced IDO1 expression and, therefore, reduced Kyn production (17). Low IDO1 activity during the hypoxic phase promotes the activation of immune cells (18); however, hypoxic conditions also augment the secretion of effector CD4(+) T-cell cytokines, especially IFN-gamma, which in turn upregulates IDO1 mRNA expression (19).

At the protein level, IDO1 is mainly regulated by proteasomal degradation in response to immunogenic stimuli. Suppressor of cytokine signaling 3 (SOCS3), for instance, binds to IDO1 under inflammatory conditions and targets the IDO1/SOCS3 complex for polyubiquitination and proteasomal digestion. IDO1 degradation converts tolerogenic dendritic cells (DCs) into immunogenic cells and, therefore, a prerequisite for normal dendritic-cell function (20). Activated AhR is another component of the ubiquitin/proteasome system that contributes to the regulatory proteolysis of IDO1 and other proteins (8) that affect IDO1’s half-life.

Indoleamine-2,3-dioxygenase 1 contains two phosphorylatable tyrosine residues (Y115 and Y253) that modulate its enzymatic activity (21). Phosphorylation of any of these residues results in conformational changes in IDO1 and blocks the catalytic activity of IDO1 (22). In addition to the regulation of catalytic activity, these motifs act as docking sites for various molecular partners that either prolong or shorten IDO1’s half-life and maintain its immunoregulatory effects or stimulate inflammatory responses, respectively (8). For example, IL-6 triggers the phosphorylation of the Y253 residue of IDO1 that recruits the ECS (Elongin-Cullin-SOCS) E3 ligase complex, targeting IDO/SOCS3 for proteasomal degradation (23). On the contrary, phosphorylation of the Y115 residue allows the binding of SH2 domain tyrosine phosphatases SHP1 and SHP2, while SOCS3 becomes excluded. Hence, the TGF-β/Fyn/SHP axis activates the non-canonical NF-κB pathway that upregulates IDO1 production. Recently, ligand-bound AhR and Arginase 1 have been also shown to promote IDO1 phosphorylation through Src kinases, activating the signaling function of IDO1 that leads to *de novo* synthesis of the enzyme *via* TGF-β production (8, 24, 25).

### The Physiological and Pathological Function of IDO1

The IDO1 pathway was originally described as an innate immune mechanism that defended the host organism against infections (26). The immunoprotective role of IDO1 was directly supported by the anti-pathogen effect of Trp metabolites (l-Kyn, l-hydroxykynurenine, 3-hydroxyanthranilic acid, quinolinic acid, picolinic acid) that prevented the proliferation and spread of intracellular pathogens (4, 27). Subsequent studies, however, identified tissue macrophages producing high levels of IDO1 upon IFN-γ stimulation that strongly inhibited the proliferation of effector T cells (28). It was also shown that accumulating Trp metabolites induced the differentiation of regulatory T cells and apoptosis of effector T cells that gave rise to immunosuppression (4, 26). l-Kyn is particularly toxic to lymphocytes (2) and induces the differentiation of regulatory T cells *via* AhR binding (29). l-hydroxykynurenine aids the suppression of CD4+ T cells and promotes the action of regulatory T cells (30). 3-hydroxyanthranilic acid modulates the immune functions of monocytes and lymphocytes, induces the apoptosis of effector T cells, and promotes the proliferation of regulatory T cells. Quinolinic acid stimulates the cell cycle of cancer cells and contributes to the acquisition of multidrug resistance against chemotherapeutic agents (29). Picolinic acid inhibits effector T cell proliferation (31). Later, it has become clear that the balance between the immunoprotective and immunosuppressive roles of IDO1 and Trp metabolites is tightly controlled by the stoichiometry of available local factors (e.g., IL-6, IL-12, CD40, IFN-γ, CTLA4, Foxo3a, IL-10, and PD-1) (26, 32). The resultant effect of these local activities modulates IDO1 expression and helps maintain global immune homeostasis and peripheral immune tolerance.

There are many pathologic diseases that are associated with increased IDO1 activity, including atherosclerosis, obesity, autoimmunity, major infections (e.g., community-acquired pneumonia, tuberculosis, listeriosis, influenza, HBV, HCV, HIV, sepsis), rejection of organ transplants, and cancer (2, 27). Originally, IDO1 has been considered as an anti-cancer molecule, proposing that increased IDO1 activity of antigen-presenting cells depletes the essential amino acid Trp from tumor cells and inhibits their growth. However, with the discovery of IDO1-mediated immunosuppressive functions, the pro-cancer activity of the enzyme has been recognized (33). IDO1 is overexpressed in more than 50% of tumors (34) that utilize IDO1-associated immunosuppressive mechanisms to promote their spread and survival (35). In cervical cancer, for instance, IDO1 shows a significantly higher mRNA transcription and protein expression level than in normal cervix, and also in comparison to other cancers (36). The extent of IDO overexpression also depends on the type of tumor and risk factors that reach patients in their life. For instance, oral squamous cell carcinoma (OSCC) was compared in never-smokers and never-drinkers (NSND) with smoker and drinker (SD) patients. In NSND patients suffering from OSCC, expression of IDO1 was significantly higher than in SDs (37).

Indoleamine-2,3-dioxygenase1 production is elevated upon (i) IFN-γ production of effector T cells (2), (ii) inflammatory cytokine production of innate immune cells (6, 38), (iii) IL-10 and IL-27 stimulation (39), (iv) CTLA4 expression on Treg cells causing increased IDO1 secretion by dendritic cells (DCs) (40), and (v) TGF-β, IL-10 and adenosine production of Treg and other immunosuppressive cells (40–42), (vi) cyclooxygenase-2 (COX-2) and prostaglandin E2 (PGE2) stimuli that are mediated through the PKC, PI3K, and MAPK pathways (several types of tumors carry PI3K or MAPK oncogenic mutations leading to constitutive IDO1 expression.) (43).

The mechanism of IDO1-elicited immunosuppression is not fully understood; however, increased IDO1 and Kyn levels are known to inhibit natural killer (NK) cell function (44, 45), prevent the activation of effector T cells, stimulate the activation of Treg cells (35, 46) and the differentiation of tolerogenic DCs (47), and promote the expansion and activation of myeloid-derived suppressor cells (48). Furthermore, Trp depletion inhibits mechanistic target of rapamycin complex 1 that stimulates T cell apoptosis and antigen-presenting cell-mediated inflammation (1, 49).

Paradoxically, the adaptive and innate immune systems that primarily protect against cancer development drive the formation of the highly aggressive and difficult-to-treat IDO1+ tumors. The genetic and biochemical characteristics of the emerging tumor is established by the process of “immunoediting” that prevents and promotes tumor formation at the same time, involving three consecutive stages called “elimination,”, “equilibrium,” and “escape” (50) (Figure 1C). In the first phase (“elimination”), most transformed cells are efficiently recognized and destroyed by the action of effector cells as NK and T cells (50). At this stage, IDO1 is produced at low levels within the tumor microenvironment and inhibits tumor proliferation (33). In the “equilibrium” phase, surviving tumor cells become “edited” by the continuous attack of the immune system and accumulate mutations (51). In the “escape” phase, IDO1 is produced in large quantities by tumor cells and tolerogenic immune cells that are recruited to the tumor microenvironment (52, 53). Increased IDO1 activity leads to elevated Kyn production that prevents effector T cell (35, 46) and NK cell functions (44, 45). In parallel, IDO1 induces the activation and expansion of Treg cells (26), DCs (47), and MDCSs (48) that further suppress the function of antitumor T cells. These mechanisms collectively establish an immunosuppressive tumor microenvironment that supports tumor growth. IDO1 positivity is strongly associated with multidrug resistance of tumors and inversely correlates with patient survival (54). Therefore, timely diagnosis and therapeutic correction of IDO+ tumors are of crucial importance to prevent clinical manifestation.

### IDO1 in Cancer Diagnostics and Therapy

Indoleamine-2,3-dioxygenase 1 overexpression increases the relative concentration of Kyn compared to Trp, hence Kyn/Trp ratio can be used as a prognostic clinico-pathological marker to monitor cancer invasiveness and progression. Accordingly, increased systemic Kyn/Trp ratio and elevated IDO1 activity have been associated with poor prognosis and low survival of patients diagnosed with cervical cancer and glioblastoma multiforme (55, 56). For the sensitive detection of Trp metabolites in IDO1^+^ tumor tissues, a wealth of Trp-based radiotracers has been developed for positron emission tomography imaging. Radioactive Trp analogs as α-[^11^C]methyl-l-tryptophan (AMT) and l- and d-1-[^18^F]fluoroethyl-tryptophan provide useful information about response to immunotherapy, but they are also crucial for the preclinical and clinical validation of novel IDO1 inhibitors (57, 58).

Protein expression of IDO1 was found to be high in a number of tumor samples (36, 56); therefore, IDO1 may be a relevant therapeutic target to abrogate immune suppression (59). Currently, several IDO inhibitors undergo clinical evaluation (60) and many of them are now in Phase II clinical trials (Table 1). Most inhibitors were designed to block the Kyn pathway (e.g., epacadostat, indoximod, GDC-0919, and an IDO1 peptide-based vaccine; Figure 1A) that suspends immunosuppression (1). Some of the tested compounds are used alone, or in combination with immunotherapy (CTLA4, PD-1 blockade), chemotherapy, adoptive transfer, COX-2 inhibitors (e.g., celecoxib), membrane-associated PGE2 synthase inhibitors (e.g., MF63), PGE2 receptor (EP4) competitive antagonists (e.g., GW627368X), and PI3K inhibitors (43, 59). The latter combinative therapies are intended to improve the inhibition of local immunosuppression around tumor tissues and to enhance tumor eradication (61).

**Table 1:** Clinical trials of indoleamine-2,3-dioxygenase (IDO) inhibitors in cancer therapy.

Agent	Tumor type	NCT number	Study phase	Recruitment status	(Estimated) primary completion date
IDO peptide vaccine	• Non-small cell lung cancer (NSCLC)	NCT01219348	Phase 1	Completed	August 2012
	• Metastatic malignant melanoma	NCT02077114	Phase 1	Completed	September 2014
		NCT01543464	Phase 2	Terminated	September 2016
		NCT03047928	Phase 1	Not yet recruiting	1 April, 2019
			Phase 2		

Indoximod (1-methyl-d-tryptophan; D-1MT; NSC-721782)	• Unspecified adult solid tumors	NCT00567931	Phase 1	Completed	July 2012
	• Breast cancer	NCT00739609	Phase 1	Terminated	October 2012
	• Lung cancer				
	• Melanoma				
	• Pancreatic cancer				
	• Solid tumors				
	• Metastatic breast cancer	NCT01302821		Withdrawn	December 2014
		NCT01792050	Phase 2	Active, not recruiting	December 2016
	• Metastatic melanoma	NCT02073123	Phase 1	Recruiting	December 2016
			Phase 2		
	• Glioblastoma multiforme	NCT02052648	Phase 1	Recruiting	December 2016
	• Glioma		Phase 2		
	• Gliosarcoma	NCT02502708	Phase 1	Recruiting	July 2017
	• Malignant brain tumor				
	• Non-small cell lung cancer (NSCLC)	NCT02460367	Phase 1	Recruiting	June 2017
			Phase 2		
	• Metastatic pancreatic adenocarcinoma	NCT02077881	Phase 1	Recruiting	July 2017
	• Metastatic pancreatic cancer		Phase 2		
	• Acute myeloid leukemia	NCT02835729	Phase 1	Recruiting	July 2018
			Phase 2		

Epacadostat (INCB024360, 4-amino-1,2,5-oxadizaole-3-carboximidamide)	• Advanced malignancies	NCT01195311	Phase 1	Completed	May 2013
	• Myelodysplastic syndromes (MDS)	NCT01822691	Phase 2	Completed	January 2015
	• Epithelial ovarian cancer	NCT01685255	Phase 2	Terminated	23 October, 2014
	• Fallopian tube cancer	NCT02118285	Phase 1	Completed	12 November, 2015
	• Primary peritoneal cancer	NCT02042430		Active, not recruiting	31 March, 2016
		NCT01982487	Phase 1	Withdrawn	September 2017
			Phase 2		
		NCT02166905	Phase 1	Recruiting	12 February, 2018
			Phase 2		
		NCT02785250	Phase 1	Recruiting	May 2018
		NCT02575807	Phase 1	Recruiting	December 2018
			Phase 2		
	• Mucosal melanoma	NCT01961115	Phase 2	Active, not recruiting	31 October, 2016
	• Skin melanoma				
	• Uveal melanoma	NCT01604889	Phase 1	Terminated	27 December, 2016
			Phase 2		
	• Gastrointestinal stromal tumors	NCT03291054	Phase 2	Not yet recruiting	September 2019
	• Recurrent/metastatic endometrial carcinoma	NCT03310567	Phase 2	Not yet recruiting	30 January, 2020
	• Squamous cell carcinoma of the head and neck	NCT03325465	Phase 2	Not yet recruiting	June 2020
	• Advanced solid tumors	NCT02559492	Phase 1	Active, not recruiting	December 2017
		NCT03085914	Phase 1	Recruiting	April 2021
			Phase 2		
		NCT02959437	Phase 1	Recruiting	September 2021
			Phase 2		
	• Metastatic pancreatic adenocarcinoma	NCT03006302	Phase 2	Not yet recruiting	February 2021
	• Metastatic non-small cell lung cancer (NSCLC)	NCT03322540	Phase 3	Not yet recruiting	17 June, 2022
		NCT03322566	Phase 3	Not yet recruiting	26 October, 2022
	• Renal cell carcinoma	NCT03260894	Phase 3	Not yet recruiting	May 2023

GDC-0919	• Advanced solid tumors	NCT02048709	Phase 1	Completed	February 2016

HTI-1090 (SHR9146)	• Advanced solid tumors	NCT03208959	Phase 1	Not yet recruiting	1 April, 2018

PF-06840003	• Oligodendroglioma • Astrocytoma • Malignant glioma	NCT02764151	Phase 1	Recruiting	30 April, 2018

NLG802	• Advanced solid tumors	NCT03164603	Phase 1	Recruiting	May 2018

BMS-986205	• Advanced cancer	NCT03335540	Phase 1	Not yet recruiting	14 March, 2021

*Clinical trials were identified on the website: https://clinicaltrials.gov/ct2/results?cond=&term=IDO&cntry1=&state1=&recrs= as of 9 November, 2017*.

Epacadostat (INCB024360) and indoximod (NLG8189 or 1-methyl-d-tryptophan) are the most common IDO inhibitors that are well tolerated and show obvious beneficial effects in cancer therapies; however, both have some major side effects. Epacadostat showed grade 3 and 4 adverse effects in patients with advanced malignancies, most frequently abdominal pain, hypokalemia, fatigue, and some minor effects involving nausea, decreased appetite, vomiting, constipation, diarrhea, dyspnea, back pain, and cough (62). Indoximod treatment also showed some major toxicities in a dose-escalation study, involving grade 1 fatigue and grade 2 hypophysitis (63). In combination with the microtubular poison docetaxel, the most common adverse effects of indoximod were fatigue, anemia, hyperglycemia, infection, and nausea (62, 63).

Combination of IFN-γ treatment with IDO1 inhibitors is a promising new cancer immunotherapeutic strategy that effectively enhances antitumor immunity and eliminates TRCs (i.e., stem cell-like cancer cells that are self-renewing, highly tumorigenic, and can repopulate tumors). In clinical practice, administration of IFN-γ with IDO1 inhibitors is the only oncolytic therapy that significantly disrupts TRCs. IFN-γ induces the entry of TRCs into dormancy, while IDO1 inhibitor-elicited immunosuppression allows effector T cells and NK cells to attack dormant TRCs (64). Monotherapies with IDO inhibitors or other combinative treatments usually terminate with failure because of immune evasion of TRCs, which leads to metastasis formation, tumor recurrence and multidrug resistance (64).

Mutational load/neoantigen-burden, basal level of tumor infiltrating T cells (TILs), differential expression of immune-checkpoint genes within the tumor tissue are important biomarkers that help predict the tumor’s predisposition toward immune-checkpoint inhibitors (ICIs) targeting IDO1, CTLA4, or PD-1 and increase the clinical success of immunotherapies. Most ICI-responsive cancers (e.g., lung and bladder cancers, melanoma) were associated with intrinsically high TIL numbers and high mutational load/neoantigen-burden, while other cancers (e.g., glioblastoma) were predicted to be ICI-resistant based on their biomarker profiles (37, 65).

Beyond the application of biomarkers and chemical inhibitors, IDO1 can be genetically targeted by genome editing tools that offer new therapeutic opportunities for cancer patients. In animal studies, genetic inhibition of IDO1 expression reactivated the antitumor immune response against IDO^+^ cancer cells and inhibited tumor growth (63). The shIDO-ST treatment, for instance, is based on a *Salmonella typhimurium* (ST) vector that codes for a small hairpin RNA targeting IDO1 (shIDO) (66). Intravenously injected shIDO-ST accumulated in the tumor tissues of mice, causing IDO1 silencing and concomitant infiltration and activation of polymorphonuclear neutrophil granulocytes (PMNs). PMNs produced reactive oxygen species that established a highly toxic microenvironment for tumor cell growth (67). A recent genomic sequencing study that combined large-scale tumor exome analysis with MHC I class prediction revealed a strong positive correlation between IDO1 expression, mutational burden, and neoantigen load in cervical cancers (36). The above studies collectively identify IDO1 as an attractive target to increase the effectiveness of cancer immunotherapies.

### Conclusion and Outlook

The mechanism of “cancer immunoediting” is the direct consequence of a T cell-dependent immunoselection process that drives the formation of IDO1+ tumors. The action of a competent immune system is, therefore, determinative for the acquisition of cancer immunogenicity. Important posttranslational control mechanisms affect the activity and half-life of IDO1 (e.g., NO, hypoxia, proteasomal degradation, phosphorylation) that should be considered in terms of cancer therapy. For instance, IDO1 inhibitors could be administered as co-therapeutic agents in the presence of redox regulators, IFN-γ, or anti-IL-6. Combining IDO1 drugs with the inhibition of specific transcription factors regulating IDO1 activity (e.g., AhR) may also improve the effectiveness and specificity of chemotherapies. Current genome editing and exome sequencing technologies offer promising new strategies to identify novel tumor-specific mutational antigens and thus expand the repertoire of tumor-specific immunotherapies.

## Author Contributions

All authors participated in the writing of this manuscript and agreed to be accountable for the content of the work.

## Conflict of Interest Statement

The authors declare that the research was conducted in the absence of any commercial or financial relationships that could be construed as a potential conflict of interest.

## Footnotes

^1^https://www.proteinatlas.org/ENSG00000151790-TDO2/tissue.

^2^https://www.proteinatlas.org/ENSG00000188676-IDO2/tissue.

^3^https://www.proteinatlas.org/ENSG00000131203-IDO1/tissue.
